# Temperature-dependent bursting pattern analysis by modified Plant model

**DOI:** 10.1186/s13041-014-0050-5

**Published:** 2014-07-22

**Authors:** Nam Gyu Hyun, Kwang-Ho Hyun, Kwang-Beom Hyun, Kyungmin Lee

**Affiliations:** 1 Department of Physics and Research Institute for Basic Sciences, Jeju National University, Jeju 690-756, South Korea; 2 School of Medicine, The Catholic University of Korea, Seoul 137-701, South Korea; 3 Department of Biological Sciences, Korea Advanced Institute of Science and Technology, Daejeon 305-701, South Korea; 4 Department of Anatomy, Brain Science & Engineering Institute, Kyungpook National University Graduate School of Medicine, 2-101, Dongin-dong, Jung-gu, Daegu 700-842, South Korea

**Keywords:** Temperature dependence, Bursting patterns, Modified Plant model, R15 pacemaker neuron, Aplysia juliana

## Abstract

Many electrophysiological properties of neuron including firing rates and rhythmical oscillation change in response to a temperature variation, but the mechanism underlying these correlations remains unverified. In this study, we analyzed various action potential (AP) parameters of bursting pacemaker neurons in the abdominal ganglion of *Aplysia juliana* to examine whether or not bursting patterns are altered in response to temperature change. Here we found that the inter-burst interval, burst duration, and number of spike during burst decreased as temperature increased. On the other hand, the numbers of bursts per minute and numbers of spikes per minute increased and then decreased, but interspike interval during burst firstly decreased and then increased. We also tested the reproducibility of temperature-dependent changes in bursting patterns and AP parameters. Finally we performed computational simulations of these phenomena by using a modified Plant model composed of equations with temperature-dependent scaling factors to mathematically clarify the temperature-dependent changes of bursting patterns in burst-firing neurons. Taken together, we found that the modified Plant model could trace the ionic mechanism underlying the temperature-dependent change in bursting pattern from experiments with bursting pacemaker neurons in the abdominal ganglia of *Aplysia juliana*.

## Introduction

To date, researchers have investigated the effect of temperature on the electrical activity and firing patterns in neurons from many animals, including *Aplysia juliana*, crabs, crayfish, frogs, lobsters, locusts, snails, and squids [[1]-[9]]. Especially the functions and properties of bursting pacemaker neuron R15 in the abdominal ganglion of *Aplysia* have been extensively studied [[10]-[12]] and mathematical simulation of bursting activity has been successfully performed [[13]-[19]]. However, few studies on the temperature dependence of action potential (AP) parameters in the R15 bursting pacemaker neuron have been reported [[20],[21]]. They analyzed typical changes of AP parameters in burst-firing neurons by investigating the effect of heat on R15 bursting pacemaker neuron activity as the temperature increased and reported temperature-dependent changes in inter-burst interval, burst duration, number of spike per burst, intra-burst spike broadening and spike height [[21]]. However, the reproducible properties and mechanism of temperature-dependent changes of AP parameters remain unknown yet.

Changes in temperature can produce numerous effects on the neural tissue of most organisms. Indeed it has been reported that hyperthermic temperature may induce depolarization and spontaneous firing of pyramidal neurons leading to enhanced excitability of hippocampus [[22],[23]]. On the other hand, a body of evidence indicating a therapeutic effect of hypothermia has accumulated in several conditions. Orlowski and colleagues published that in refractory status epilepticus unresponsive to conventional treatment, systemic hypothermia (30-31°C) was an effective therapeutic method leading to burst suppression on electroencephalography (EEG) [[24]]. Despite a lot of works aiming at demonstrating the neural effect of temperature, the mechanism of temperature-dependent electrophysiological change containing alteration of bursting and firing pattern in neuron remains unclear. Here, we examined the effects of temperature changes on the neuronal activity and bursting patterns during several consecutive heating − cooling cycles by using bursting pacemaker neurons which are a proper specimen with capability of long-lasting recording for mathematical modeling. Next, we sought to identify the mechanism underlying temperature-dependent bursting patterns of these neurons by analyzing and comparing the experimental data to computational simulation data calculated by modified Plant equations with temperature-dependent scaling factors, ρ(T) and ϕ(T).

## Materials and methods

### Animals and dissection

Animals (*A. juliana*) were collected locally in Seogwipo, South Korea and all experimental procedures were approved by the Jeju National University Animal Care and Use Committee. These animals were dissected to observe the temperature-dependency of bursting patterns generated by pacemaker neurons R15 in the abdominal ganglia of *A. juliana* as described in previously published paper [[1]]. Briefly, the animals were anesthetized with an injection of 0.38 M MgCl_2_ amounting to half of each specimen’s weight before the abdominal ganglia were removed. Each abdominal ganglion was incubated at 34°C for 40 minute in a solution containing equal volumes of artificial sea water (ASW; in mM: 460 NaCl, 10 KCl, 11 CaCl_2_ , 55 MgCl_2_ , and 10 HEPES; pH 7.6) and isotonic Leibovitz’s L-15 media (Cat. No. L-4386; Sigma) containing 1% protease (type IX; Sigma) (hereafter, ASW:L-15(1:1)). They were then washed several times with ASW and were placed in a low-temperature incubator (VS-1203PIN; Hanback Co., Daejeon, Korea) at 18°C. At last they were removed onto Petri dishes (50x9 mm), pinned down on Sylgard plates (Dow Corning, USA) filled with ASW:L-15(1:1), and desheathed.

### Data acquisition and analysis

A PT100 platinum resistance temperature sensor connected to a digital thermometer (TRM-006, Toho, Japan) was placed near the abdominal ganglia of *Aplysia* soaked in ASW:L-15 (1:1, v/v) to measure the medium temperature, which could be increased or decreased by activating the temperature controller system (HMN 3940, Acetec Co., Korea). We used a low flow rate peristaltic pump (BJ100-2 J; Baoding Longer Precision Pump Co., China) to maintain a viable solution with ASW:L-15 (1:1) media. The flow rate and the speed were 0.14 mL/min and 1.0 rpm, respectively. Intracellular recordings of bursting pacemaker neurons were performed to measure the membrane potential. The glass intracellular electrodes were filled with 3 M KCl and the membrane potentials recorded were simultaneously saved using a DAQ card (NI PCI-6221, National Instruments) and the Labview program (National Instruments). The electrical signals of the APs were identified using a digital oscilloscope (54622A; Agilent, Colorado Springs, Colo., USA). These data were recorded at a rate of 3 kHz to reproduce the APs on the computer because the bursting cells generated APs at a rate of 0–4 Hz; each component of this data is composed of 180,000 pairs of temperature and membrane potential values with unique file names, and the dataset was composed of 1,713 files as shown in Table 1. With this selected dataset, the average values of each of the AP parameters of the burst-firing neuron could be calculated using Origin 6.0 (Microcal Software, Inc.) and a computer program we designed. For each experiment, the bursting neuron was held at room temperature for one hour as shown in Table 2 and then changed with continuous ramp from about 16°C to 30°C. Then, the data recorded by increments (or decrements) of 2°C from about 16°C to 30°C (16-18-20-22-24-26-28-30°C) were averaged over. To reduce experimental variability, in each experiment the bursting neuron was incubated at least 5 min to adapt to a new steady-state temperature before recording rhythmic change.

**Table 1:** **Animal weights and electrophysiological properties of signals generated by eight R15 bursting pacemaker neurons in** ***A. juliana*** **specimens**

**Exp.**	**Animal weight (g)**	**Data selected for analysis**				**Total number of spikes**	**Total recording time (min)**
		**Range of temperature change (°C)**	**Time interval for analysis (min)**	**Number of tem. change cycle**	**Number of spikes**		
A	150	16.5-30.0	231	2(8)	7,861	31,890	980
B	130	16.7-29.8	230	2(6)	9,030	25,890	794
C	190	14.5-30.8	210	2(3)	3,640	6,230	491
D	210	14.4-29.5	137	1(1)	4,776	9,121	292
E	290	13.5-30.3	225	2(4)	6,670	18,668	623
F	255	17.3-32.5	228	2(5)	3,613	10,500	674
G	310	14.4-30.9	225	2(4)	3,884	10,168	570
H	280	15.8-30.0	227	2(3)	3,994	7,848	433
Total	-	-	1,713	15(33)	43,468	120,315	4,857
Average	226	15.3-30.4	214	2(4)	5,433	15,039	607

Exp., Experiments; temp., temperature; ( ), total number of temperature change cycle.

**Table 2:** Temperature and selected AP parameters in bursting pacemaker neurons maintained for 30 min

**Experiments**	**Temperature**	**Number of bursts/min**	**Number of spikes/burst**	**Interbust interval(s)**	**Burst duration(s)**
A	21.48 ± 0.03	4.86 ± 0.31	9.49 ± 0.80	8.72 ± 0.88	2.77 ± 0.20
B	22.61 ± 0.01	6.33 ± 0.36	8.23 ± 0.53	5.96 ± 0.66	3.10 ± 0.16
C	19.45 ± 0.00	2.33 ± 0.08	9.10 ± 0.17	19.46 ± 0.47	2.01 ± 0.03
D	16.34 ± 0.03	4.63 ± 0.19	7.71 ± 0.31	9.07 ± 0.33	2.95 ± 0.08
E	19.11 ± 0.02	4.16 ± 0.17	20.82 ± 0.71	8.68 ± 0.65	5.01 ± 0.10
F	22.20 ± 0.04	3.13 ± 0.22	6.02 ± 0.38	14.47 ± 0.96	3.56 ± 0.16
G	18.22 ± 0.03	2.43 ± 0.13	8.64 ± 0.19	18.79 ± 0.63	2.37 ± 0.05
H	18.86 ± 0.02	5.90 ± 0.38	5.28 ± 0.22	8.54 ± 0.73	1.73 ± 0.07
Average	19.78 ± 0.76	4.22 ± 0.53	9.41 ± 1.70	11.71 ± 1.82	2.93 ± 0.36

## Results

### Definition of AP parameters in burst-firing neuron

**Figure 1:**
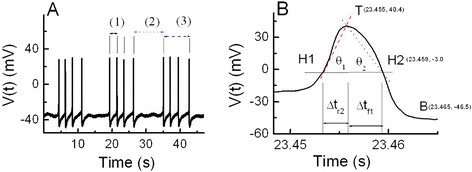
**Typical bursting patterns and AP parameters of bursting pacemaker neuron.(A)** Bursting patterns of bursting pacemaker neuron; membrane potential vs. time. The electrophysiological parameters related to burst firing contain the intraburst interspike interval, the duration between the two points of sequential V_pp_ within one burst firing event [**A** (1)]; interburst interval, the duration between the two points of the last V_pp_ of one burst firing event and the first V_pp_ of next burst firing event [**A** (2)]; and burst duration, the duration between the two points of the first V_pp_ and the last V_pp_ within one burst firing event [**A** (3)]. **(B)** Definition of AP parameters. The maximum point T on the upper part of AP means a positive peak membrane potential, V_pp_ and the minimum point B on the lower part of AP means a negative peak membrane potential, V_np_. H1 and H2 means the half point of potential difference during the rising phase and the falling phase of AP, respectively. The last half of the rising phase, Δt_r2_ designate the time intervals between the two point H1 and T. The first half of the falling phase of AP, Δt_f1_ is defined as the time interval between point T and point H2. The angle θ_1_ shows the inverse tangent of the ratio of half A_AP_ to Δt_r2_ and angle θ2 means the inverse tangent of the ratio of half A_AP_ to Δt_f1_.

The graph in Figure 1A represents the typical bursting patterns of electrical signals shown in *Aplysia* bursting pacemaker neuron. The intraburst interspike interval, interburst interval, and burst duration are represented by symbols (1), (2), and (3), respectively. The membrane potentials at the positive peak, V_pp_ (mV), and negative peak, V_np_ (mV), are defined as the values of membrane potentials at points T and B shown in Figure 1B, respectively. The definition of the first half of the rising phase of AP, Δt_r1_ (ms), and the following AP parameters are very similar to those defined in our previous study [[1]]. The last half of the rising phase of AP, Δt_r2_ (ms) and the first half of the falling phase of AP, Δt_f1_ (ms) are time intervals shown in Figure 1B. The last half of the falling phase Δt_f2_ (ms) is defined as the values of the time interval between two points H2 and B shown in Figure 1B. The AP half-width duration, Δt_AP, 1/2_ (ms), is defined as Δt_r2_ + Δt_f1_ (ms). The interspike interval, ISI (ms), is defined as Δt_r1_ + Δt_AP, 1/2_ + Δt_f2_. The spontaneous firing frequency, simply referred to as Frequency (s^−1^), is defined as ISI^−1^ (s^−1^). For convenience, the average angles θ_1_ and θ_2_ are defined to compare the slope of the second half of the rising phase of the AP with that of the first half of the falling phase. The angle θ_1_ is defined as the inverse tangent of the ratio of half A_AP_ to Δt_r2_, and the angle θ_2_ is also defined as the inverse tangent of the ratio of half A_AP_ to Δt_f1_: $\theta_{1}\equiv \mathrm{Arctan}\;\left[\frac{V\left(t_{T}\right)‐V\left(t_{H1}\right)}{t_{T}‐t_{H1}}\right]$ and $\theta_{2}\equiv \mathrm{Arctan}\;\left[\frac{V\left(t_{T}\right)‐V\left(t_{H2}\right)}{t_{H2}‐t_{T}}\right]$. Although angles are usually defined as dimensionless quantities, these angles are defined differently for convenience. The AP amplitude, A_AP_ (mV), is defined as the absolute value of difference between the two values of membrane potentials at points T and B, respectively. More detailed analytical techniques regarding the definition and computational analysis of AP parameters and others were described previously [[1],[25]]. Data were processed using the scientific data analysis tools, C^++^, Origin 6.0, and Mathematica 5.1, and the resulting data were presented as mean ± SE (standard error).

### Structure of the selected dataset for AP parameter analysis

Eight experiments (A − H) were conducted using *A. juliana* specimens, and Table 1 shows the animal weights, selected data for analysis, total number of spike, and total recording time.

Animal weights were between 130 and 310 g, and the average weight was 226 g. The average total number of spike and average total recording time of each experiment were 15,039 spikes and 607 min, respectively. It was necessary to select the middle portion of each recorded dataset for analysis. The average values of time intervals for analysis, numbers of cycles of temperature change, and number of spike selected for analysis of these selected data were 214 min, 2 cycles, and 5,433 spikes, respectively. The dataset gathered for analysis was composed of these selected data from each experiment. The total time interval for analysis and the total number of spike of this dataset were 1,713 minutes and 43,468 spikes, respectively. The numbers written in parentheses in the middle column of Table 1 represent the total number of cycles of temperature change for each experiment.

In each experiment, the temperature was maintained at room temperature for the first one hour. As shown in Table 2, average values of temperature and four AP parameters of burst-firing neurons were calculated using the selected dataset saved by intracellular recordings during maintaining at room temperature for 30 minutes; all data were selected after 5 to 10 minutes from temperature change onset. Because the standard errors of the values of these parameters were low, the bursting patterns of these experiments might be considered as regular while the temperatures were held constant at room temperature.

### Temperature dependence of AP parameters in burst-firing neurons

**Figure 2:**
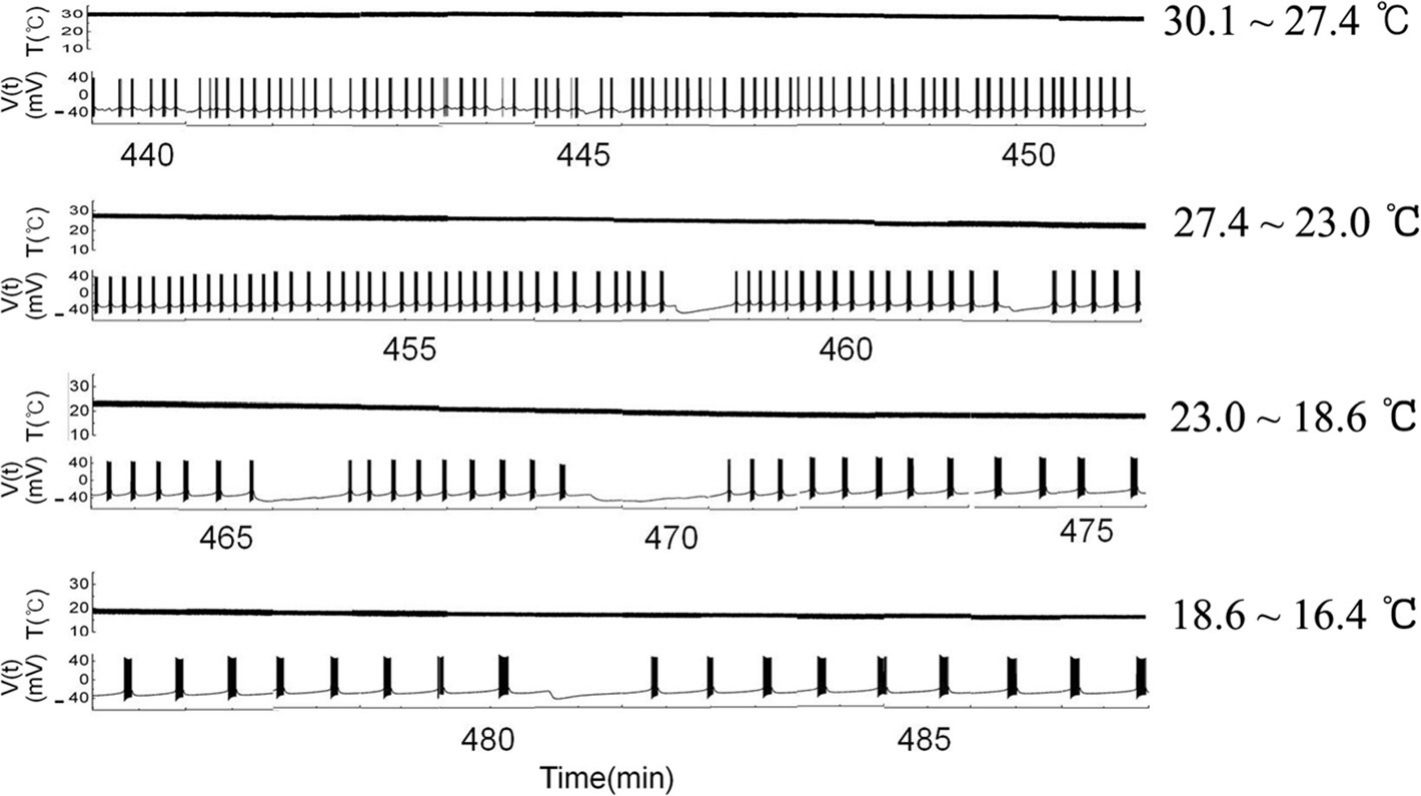
**Continuous bursting patterns obtained from the dataset of intracellular recordings of experiment A during the 4th falling phase (from 440 to 487 minutes) out of eight recorded falling phases.** The temperature values of each panel is as follows; 30.1 ~ 27.4°C (top), 27.4 ~ 23°C (2nd from the top), 23 ~ 18.6°C (3rd from the top), and 18.6 ~ 16.4°C (bottom). As temperature decreased, the interburst interval, burst duration, and number of spike during burst increased.

**Figure 3:**
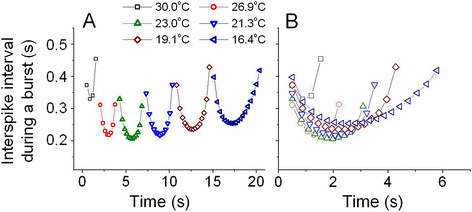
**Plot of interspike intervals during a burst versus time. (A)** Figures drawn by using data calculated from the burst shown on panels in Figure 2: parabolic shape. The values of temperature corresponding to the figures from the left to the right are 30.0°C, 26.9°C, 23.0°C, 21.3°C, 19.1°C, and 16.4°C, sequentially. **(B)** The values of each figure are the same as those shown in (A): all the figures were shown with the same start time.

We selected burst trains in a continuous time series from the intracellular recording data of experiment A during the fourth falling phase (from 440 to 487 min) (Figure 2) out of the eight falling phases in temperature recorded during eight consecutive heating − cooling cycles: it was composed of 48 panels. Temperature and membrane potentials are represented as the upper and lower traces in Figure 2, respectively. These figures demonstrated that interburst interval, burst duration, and number of spike during burst decreased as temperature increased. Next, we extracted six burst signals (6 panels) among many burst signals to examine the temporal change of instantaneous ISI during burst at specific temperature; 30.0°C (at 440 min), 26.9°C (at 454 min), 23°C (at 464 min), 21.3°C (at 468 min), 19.1°C (at 474 min), and 16.4°C (487 min). As shown in Figure 3, ISI during burst versus time displayed parabolic pattern, suggesting that temperature-dependent bursting patterns are parabolic bursts characterized by lower spike frequency at the beginning and end of the burst. The analyzing dataset was composed of 1,713 files corresponding to the sum of each selected time interval for analysis as shown in the 4th column of the Table 1.

**Figure 4:**
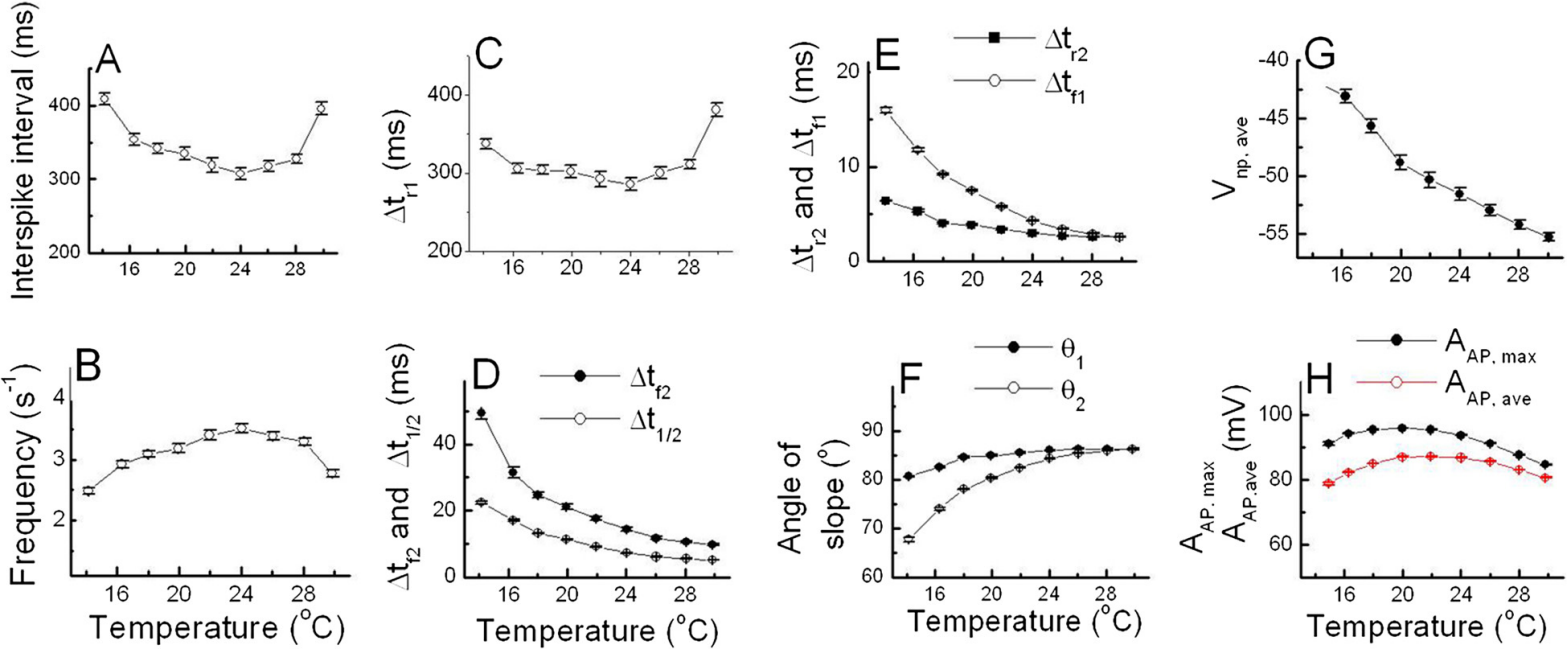
**Temperature-dependent AP parameters of burst-firing neurons.** The values of ISI **(A)** and Δt_r1_**(C)** decreased and then increased as temperature increase, but frequency **(B)** and the values of A_AP, max_ and A_AP, ave_**(H)** showed the reversed pattern according to the change of temperature. Δt_f2_ and Δt_1/2_**(D)**_,_ Δt_r2_ and Δt_f1_**(E)**, V_np,ave_**(G)** decreased as temperature increase and the values of angle θ_1_ and θ_2_**(F)** showed the reversed manner.

With the selected data shown in Table 1, the temperature-dependent properties of AP parameters were analyzed by using techniques similar to those described in Hyun et al. [[1]]. As shown in Figure 4A and C, all values of ISI and Δt_r1_ decreased and then increased as the temperature was raised, but frequency shown in Figure 4B rose and then fell as the temperature increased. Figure 4D, E and G show that the parameters Δt_f2_, Δt_1/2_, Δt_r2_, Δt_f1_, and V_np,ave_ decreased as the temperature was raised with small standard errors for each value. On the other hand, the values of angles shown in Figure 4F increased as the temperature was raised, and the values of angle θ_1_ were larger than those of θ_2._ In addition, both A_AP, max_ and A_AP, ave_ shown in Figure 4H increased and then decreased as the temperature was raised. These temperature dependencies of AP parameters (except A_AP, max_, A_AP, ave_, and angles) were similar to the case of those analyzed using a dataset obtained from beating cells: in that case, A_AP,ave_ decreased as the temperature was raised between 16°C and 28°C, but A_AP, ave_, and angles were not shown [[1]].

### Temperature dependence of bursting patterns in burst-firing neurons

**Figure 5:**
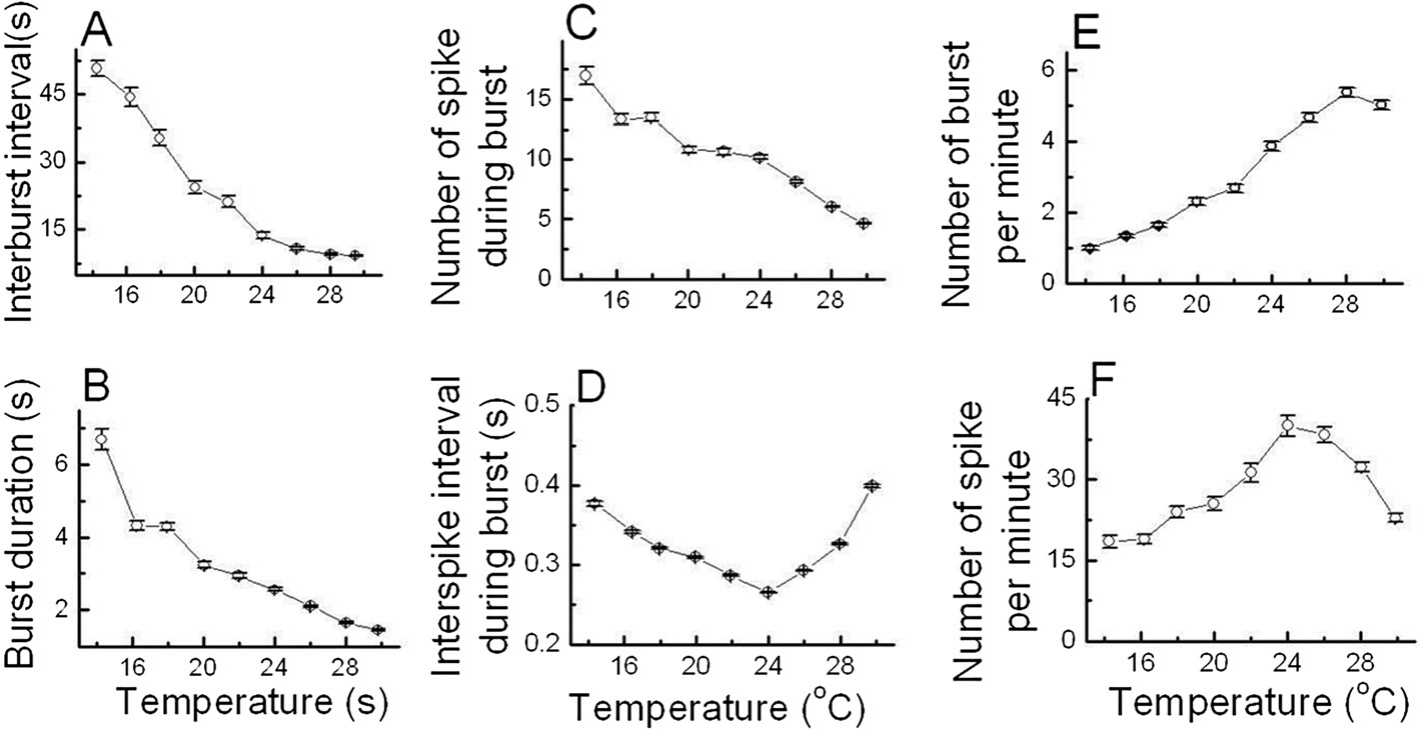
**Temperature-dependent bursting patterns of burst-firing neurons.** Interburst interval **(A)**, burst duration **(B)**, number of spike during burst **(C)** decreased as temperature increased. ISI during burst **(D)** showed a parabolic pattern which means that it decreased and then increased as temperature increased. Number of burst per minute **(E)** and number of spike per minute **(F)** increased and then decreased showing the reversed pattern of ISI during burst as temperature increased. The instantaneous ISI during a burst versus time firstly decreased and then increased showing the parabolic bursting pattern at each temperature ranging from 30°C to 16°C **(G)**.

The values of six temperature-dependent burst parameters were averaged by using these selected dataset shown in Table 1. As shown in Figure 5A, B, and C respectively, interburst interval, burst duration, and number of spike during burst decreased as temperature increased. ISI during burst shown in Figure 5D firstly decreased and then increased as temperature increased. In contrast, the numbers of burst per minute and of spike per minute shown in Figure 5E and F respectively increased and then decreased as the temperature was raised.

However, since these six burst parameters were not independent parameters, some equations representing an interrelationship between these parameters of burst-firing neurons could be driven; number of spike during burst = (burst duration)/(inter spike interval during burst), number of spike per minute = (number of spike during burst) × (number of burst per minute), and number of burst per minute = 60/(interburst interval + burst duration).

**Figure 6:**
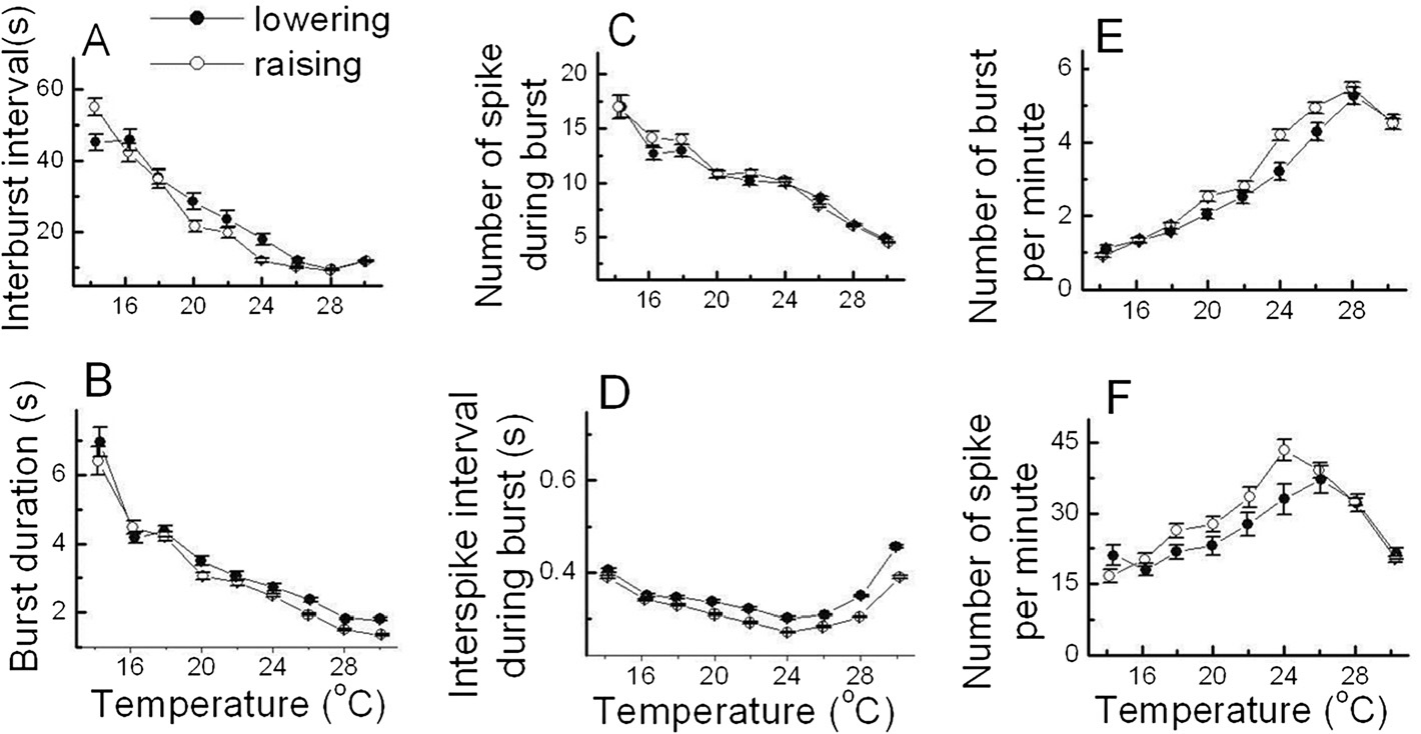
**Reproducible temperature-dependent bursting patterns of burst-firing neurons while decreasing (●) and increasing the temperature (○).** There is no remarkable difference in each AP parameters when compared the values according to temperature between the cooling-off and heating period which means the reproducible properties of temperature-dependent burst parameters (**A** to **F**).

The values of these six temperature-dependent burst parameters during lowering the temperature (●) and raising the temperature (○) are shown in Figure 6. In the cases of interburst interval, burst duration, number of spike during burst, and ISI during burst shown in Figure 6A, B, C, and D respectively, there were no significant differences in these parameter values between the cooling-off and heat periods, which could be considered that there were reproducible properties of temperature dependencies in these burst parameters. Moreover, the number of burst per minute and spike per minute during heating shown in Figure 6E and F were slightly larger than those during cooling between 19°C and 25°C. However, it might be thought that the reproducible property of temperature-dependent changes in bursting patterns could not be undermined by these. It is because the values of the experimental percentage error of interspike interval during burst were between 0.1% and 14.1%; experimental percentage error ≡ 100% × | value of bursting parameter during heating (cooling) - average value of bursting parameter |/ average value of bursting parameter.

Thus, each mean value of functional forms of reproducible temperature-dependent bursting parameters in Figures 5 and 6 could be calculated by using these dataset and the computer program we designed. It was shown that these six bursting parameters changed in response to temperature variation, but the mechanism underlying these correlations remains unverified. So, it was suggested that performing computational simulations of these phenomena by using a modified Plant model which were composed of equations with temperature-dependent scaling factors was necessary to mathematically clarify the temperature-dependent changes of bursting patterns in burst-firing neurons.

### Simulation of temperature-dependent bursting patterns

In order to simulate various temperature-dependent spiking patterns of bursting pacemaker neuron, we set up nonlinear differential equations by modifying the Plant model [[15]] with temperature-dependent scaling factors [[25]-[27]]. The Plant model studied by Rinzel and Lee was designed to assess parabolic bursters by analyzing the fast and slow processes to show how spike activities were generated by their mutual interaction [[16]].

The cell membrane of modified Plant model contains sodium channels carrying a fast sodium current, I_Na_, and potassium channels underlying a fast potassium current, I_K_. The slow processes include the slow inward calcium current, I_Ca_, and the slow changes in intracellular free calcium concentration, Ca. The accumulation of calcium turns on outward calcium-activated potassium current, I_K(Ca)_, and undermines the slow inward calcium current, and finally brings to an end of bursting activity. This system also contains a leak current, I_L_.

(1) $$\begin{array}{c} -C_{m}\frac{\mathrm{dV}}{\mathrm{dt}}=\;\rho \left(T\right)\;\left(I_{\mathrm{Na}}+I_{\mathrm{Ca}}+I_{K}+I_{K\left(\mathrm{Ca}\right)}\right)+I_{L}\\ =\rho \left(T\right)\left\{{\bar{g}}_{\mathrm{Na}}\cdot m_{\infty }^{3}\left(V\right)\cdot h\cdot \left({V‐V}_{\mathrm{Na}}\right)+{\bar{g}}_{\mathrm{Ca}}\cdot \chi \cdot \left({V‐V}_{\mathrm{Ca}}\right)+\left({\bar{g}}_{K}\cdot n^{4}+{\bar{g}}_{K‐\mathrm{Ca}}\frac{\mathrm{Ca}}{0.2+\mathrm{Ca}}\right)\cdot \left({V‐V}_{K}\right)+{\bar{g}}_{L}\cdot \left({V‐V}_{L}\right),\right) \end{array}$$

(2) $$\begin{array}{c} \frac{\mathrm{dh}}{\mathrm{dt}}=\phi \left(T\right)\cdot \lambda \frac{\left[h_{\infty }\left(V\right)‐h\right]}{\tau_{h}\left(V\right)},\\ \frac{\mathrm{d\chi}}{\mathrm{dt}}=\phi \left(T\right)\frac{\chi_{\infty }\left(V\right)-\chi }{\tau \chi },\\ \frac{\mathrm{dn}}{\mathrm{dt}}=\phi \left(T\right)\cdot \lambda \frac{\left[n_{\infty }\left(V\right)-n\right]}{\tau_{n}\left(V\right)},\\ \frac{\mathrm{dCa}}{\mathrm{dt}}=\rho \cdot \left[K_{c}\cdot \chi \cdot \left(V_{\mathrm{Ca}}-V\right)-\mathrm{Ca}\right], \end{array}$$

Where

(3) $$\begin{array}{c} m_{\infty }\left(V\right)=\frac{\mathrm{\mu m}\frac{50-\mathrm{\alpha V}-\beta }{e^{\left(\frac{50-\mathrm{\alpha V}-\beta }{10}\right)}-1}}{\mathrm{\mu m}\frac{50-\mathrm{\alpha V}-\beta }{e^{\left(\frac{50-\mathrm{\alpha V}-\beta }{10}\right)}-1}+4e^{\left(\frac{25-\mathrm{\alpha V}-\beta }{18}\right)}},\\ h_{\infty }\left(V\right)=\frac{\mu_{h}\;e^{\left(\frac{25-\mathrm{\alpha V}-\beta }{20}\right)}}{\mu_{h}\;e^{\left(\frac{25-\mathrm{\alpha V}-\beta }{20}\right)}+\frac{1}{e^{\left(\frac{55-\mathrm{\alpha V}-\beta }{10}\right)}}},\\ \chi_{\infty }\left(V\right)=\frac{1}{1+e^{\gamma \left(\delta -V\right)}}, \end{array}$$

and

(4) $$\begin{array}{c} n_{\infty }\left(V\right)=\frac{\mathrm{\mu n}\frac{55-\mathrm{\alpha V}-\beta }{e^{\left(\frac{55-\mathrm{\alpha V}-\beta }{10}\right)}}}{\mathrm{\mu n}\frac{55-\mathrm{\alpha V}-\beta }{e^{\left(\frac{55-\mathrm{\alpha V}-\beta }{10}\right)}-1}+\nu_{n}\;e\left(\frac{45-\mathrm{\alpha V}-\beta }{80}\right)},\\ \tau_{h}\left(V\right)=\frac{1}{\mu_{h}\;e^{\left(\frac{25-\mathrm{\alpha V}-\beta }{20}\right)}+\frac{1}{e^{\left(\frac{55-\mathrm{\alpha V}-\beta }{10}\right)}+1}}, \end{array}$$

and

(5) $$\tau_{n}\left(V\right)=\frac{{\bar{\tau }}_{n}}{\mu_{n}\frac{55-\mathrm{\alpha V}-\beta }{e^{\left(\frac{55-\mathrm{\alpha V}-\beta }{10}\right)}-1}+\nu_{n}\;e^{\left(\frac{45-\mathrm{\alpha V}-\beta }{80}\right)}}$$

Where ${\bar{g}}_{\mathrm{Na}}$, ${\bar{g}}_{\mathrm{Ca}}$, ${\bar{g}}_{K}$, and ${\bar{g}}_{L}$ are maximal conductances for the Na^+^, Ca^2+^, K^+^, and Cl^−^ currents, respectively, and V_Na_, V_Ca_, V_K_, and V_L_ are the reversal potentials for the respective currents: ${\bar{g}}_{K\left(\mathrm{Ca}\right)}$ is maximal conductance for calcium-activated potassium current. Here, voltage, V indicates membrane potential (mV).

The voltage-dependent activation and inactivation variables for sodium channels are *m* and *h* respectively, and the activation variable for potassium channels is *n*. A slowly activating conductance for calcium current is denoted by χ, and a slow change in intracellular free calcium concentration Ca, is treated as parameters. The maximal relaxation time constants of h and n are defined as $\frac{1}{\lambda }$. It is taken that ρ^−1^ is an estimate for the time-constant of the Ca-equation. The temperature-dependent scaling factors, ρ(T) and ϕ(T), are defined as $\rho \left(T\right)\equiv 1.3^{\frac{{T‐T}_{0}}{10\;{}^{\circ}C}}$ and $\phi \left(T\right)\equiv 3^{\frac{T-T_{0}}{10{}^{\circ}C}}$, respectively [[25]]. The steady-state values of activation or inactivation variables m_∞_, h_∞_, χ_∞_, and n_∞_ are functions of voltage. The relaxation time constants are represented by τ_h_, τ_χ_, and τ_n_.

### Comparison of experimental and simulation results

**Figure 7:**
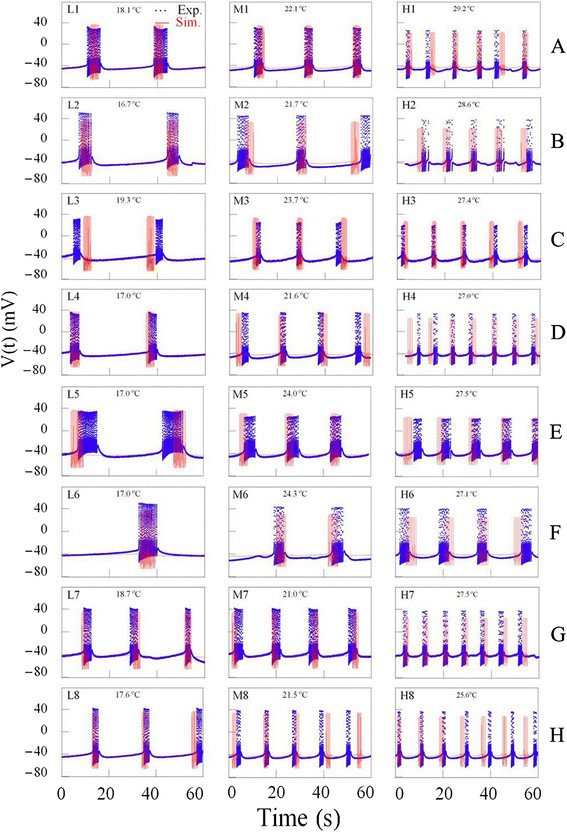
**Time series of bursting activity drawn by using the data for the experiments from A to H and computer simulations (A to H from top to bottom panel).** The results of experiments (blue, dotted line) and computer simulation (red, solid line) are represented as the activity with the same corresponding temperatures shown on each panel. The figures shown by using data files selected from datasets of experiments **A** – **H** (from top to bottom panel) were symbolized by L1- L8, M1 - M8, and H1 - H8, at the temperature ranges below 20°C, between 20°C and 25°C, above 25°C, respectively. Computer simulations should be carried out until percentage errors of all parameters had to be calculated below 50%.

In order to obtain good computer simulation of temperature-dependent bursting patterns generated respectively by these eight bursting pacemaker neurons, it was necessary to select three good data files of each experiment. The figures drawn by using data files selected during the same rising (or falling) phase of temperature change from dataset of experiment A at temperatures below 20°C, between 20°C and 25°C, above 25°C, were expressed by symbols L1, M1, H1, respectively, shown on Figure 7. Similarly, the figures shown by using data files selected from datasets of experiments B – H at the same temperature ranges were symbolized by L2- L8, M2 - M8, and H2 - H8, respectively, shown on Figure 7.

Then, it was necessary to describe how to draw three figures shown on the panels L1, M1, and H1 in Figure 7. Firstly, temperature values shown on these figures should be substituted for T in the temperature-dependent scaling factors, ρ(T) and ϕ(T) involved in modified Plant model. Secondly, computer simulation should be carried out until percentage errors of all parameters had to be calculated below 50%: the results of these calculations were shown at first three rows in Table 3. However, percentage error could be defined with the following formula shown below:

(6) $$\begin{array}{l} \mathrm{Percentage}\;\mathrm{error}\;\equiv \\ \frac{\left|\;\mathrm{the}\;\mathrm{average}\;\mathrm{value}\;\mathrm{of}\;\mathrm{the}\;\mathrm{simulated}\;\mathrm{data}\;‐\mathrm{the}\;\mathrm{average}\;\mathrm{value}\;\mathrm{of}\;\mathrm{the}\;\mathrm{experimental}\;\mathrm{data}\;\right|}{\mathrm{the}\;\mathrm{average}\;\mathrm{value}\;\mathrm{of}\;\mathrm{the}\;\mathrm{experimental}\;\mathrm{data}}\;\times \;100\;\% \end{array}$$

**Table 3:** Comparison of experimental and simulation results

**Exp**	**Parameters of ** **modified Plant model**			**Burst parameters and percent error**														
				**Number of burst per minute**			**Number of spike during burst**			**Interburst interval (s)**			**Burst duration (s)**			**Interspike interval during burst (ms)**		
	**ρ**	**τ**_***x***_	**T (°C)**	**Ex**	**Si**	**Err (%)**	**Ex**	**Si**	**Err (%)**	**Ex**	**Si**	**Err (%)**	**Ex**	**Si**	**Err (%)**	**Ex**	**Si**	**Err (%)**
A	0.000074	1500	18.1	20	20	0.0	21.0	13.0	38.0	22.5	23.5	4.4	5.2	3.4	34.6	247	261	5.6
			22.1	3.0	3.0	0.0	16.0	12.0	25.0	15.5	17.3	11.6	3.2	3.1	3.1	200	258	29.0
			29.2	6.0	6.0	0.0	5.0	7.0	40.0	7.6	7.8	2.6	1.3	1.9	46.1	260	271	4.2
B	0.00015	9000	16.7	20	20	0.0	11.0	13.0	18.1	32.0	31.6	1.2	4.8	4.2	12.5	436	323	25.9
			21.7	3.0	3.0	0.0	11.0	11.0	0.0	16.0	18.9	18.1	3.9	3.2	17.9	354	290	18.0
			28.6	5.0	5.0	0.0	5.0	7.0	40.0	8.2	8.0	2.4	2.3	2.8	21.7	460	400	13.0
C	0.00006	790	19.3	20	20	0.0	11.0	13.0	18.1	31.7	23.3	26.4	2.6	3.2	23.0	236	246	4.2
			23.7	3.0	3.0	0.0	8.0	11.0	37.5	13.8	15.9	15.2	1.9	2.4	26.3	237	218	8.0
			27.4	5.0	5.0	0.0	7.0	8.0	14.2	9.6	10.1	5.2	1.2	1.7	41.6	171	212	23.9
D	0.00028	13000	17.0	20	20	0.0	13.0	10.0	23.0	29.0	28.0	3.4	3.4	3.2	5.8	261	320	22.6
			21.6	4.0	4.0	0.0	9.3	8.0	13.9	13.0	15.2	16.9	2.2	2.5	13.6	236	312	32.2
			27.0	7.0	7.0	0.0	4.8	5.0	4.1	6.4	6.9	7.8	1.3	1.9	46.1	270	380	40.7
E	0.00015	15000	17.0	20	20	0.0	27.0	15.0	44.4	27.9	38.8	39.0	7.7	4.9	36.3	285	326	14.3
			24.0	3.0	3.0	0.0	18.0	11.0	38.8	11.6	16.5	42.2	4.5	3.5	22.2	250	318	27.2
			27.5	5.0	5.0	0.0	12.5	8.2	34.4	8.8	10.4	18.1	3.4	3.3	2.9	272	402	47.7
F	0.00016	27000	17.0	1.0	1.0	0.0	18.0	18.0	0.0	(69.3)	(50.9)	(26.5)	7.3	6.0	17.8	405	333	17.7
			24.3	20	20	0.0	10.0	11.0	10.0	19.7	18	8.6	3.9	3.9	0.0	390	354	9.2
			27.1	4.0	4.0	0.0	12.6	10.0	20.6	10.4	11.5	10.5	3.7	3.8	2.7	293	380	29.6
G	0.00022	7000	18.7	3.0	3.0	0.0	10.5	9.0	14.2	15.3	18.8	22.8	3.0	2.7	10.0	285	300	5.2
			21.0	4.0	4.0	0.0	13.3	8.0	39.8	10.0	14.4	44	3.3	2.5	24.2	248	312	25.8
			27.5	7.0	7.0	0.0	6.0	5.0	16.6	5.9	6.6	11.8	1.5	1.6	6.6	250	320	28.0
H	0.00028	7000	17.6	3.0	3.0	0.0	7.0	8.0	14.2	17.6	18.7	6.2	2.1	2.5	19.0	300	312	4.0
			21.5	5.0	5.0	0.0	6.0	6.0	0.0	8.8	11.6	31.8	1.3	1.6	23.0	216	266	23.1
			25.6	7.0	7.0	0.0	5.0	5.0	0.0	7.4	7.3	1.3	1.0	1.4	40.0	200	280	40.0

(): 2-minute average value, Exp & Ex: experimental value.

Si: simulation parameter value, $\mathrm{Err}.\;\mathrm{Percent}\;\mathrm{error}\;=\;\frac{\left|\;\mathrm{the}\;\mathrm{average}\;\mathrm{of}\;\mathrm{the}\;\mathrm{experimental}\;\mathrm{data}‐\;\mathrm{the}\;\mathrm{average}\;\mathrm{of}\;\mathrm{the}\;\mathrm{simulated}\;\mathrm{data}\;\right|}{\mathrm{the}\;\mathrm{average}\;\mathrm{of}\;\mathrm{the}\;\mathrm{experimental}\;\mathrm{data}}\times \;100\;\%$. Computer simulation should be carried out by changing parameter values ρ and τ_x,_ until percentage errors of all parameters had to be calculated below 50%.

Finally, numerical values of each of the 24 parameters involved in equations (1) to (4) would be fixed: C_m_ = 1 μF/cm^2^, ${\bar{g}}_{\mathrm{KCa}}=0.018\;\mathrm{mmho}/{\mathrm{cm}}^{2}$, ${\bar{g}}_{\mathrm{Na}}=4.0\;\mathrm{mmho}/{\mathrm{cm}}^{2}$, ${\bar{g}}_{\mathrm{Ca}}=0.007\;\mathrm{mmho}/{\mathrm{cm}}^{2}$, ${\bar{g}}_{K}=0.60\;\mathrm{mmho}/{\mathrm{cm}}^{2}$, ${\bar{g}}_{L}=0.017\;\mathrm{mmho}/{\mathrm{cm}}^{2}$, V_Na_ = 40 mV, V_Ca_ = 140 mV, V_K_ = −75 mV, V_L_ = −40 mV, λ = 0.18, ρ = 0.000074 ms^−1^, τ_χ_ = 1, 500, K_c_ = 0.0275 mV^−1^, α = 127/105, β = 8265/105, γ = 0.3, δ = −18, μ_m_ = 0.1, μ_h_ = 0.08, μ_n_ = 0.016, ν_n_ = 0.1, ${\bar{\tau }}_{n}=1$, T_0_ = 23.0°C. Here, we took a value of 23°C as the reference temperature T_0_ because bursts are usually activated from 22°C to 25°C, and this represented the middle value of temperature range of the experiment A; from 16.0°C to 30.0°C.

To simulate the other figures shown in Figure 7, it was necessary to fix the same numerical values of each of the 22 parameters as those used in experiment A, except the values of ρ and τ_x_. And then, computer simulation should be carried out by changing parameter values ρ and τ_x_ until percentage errors of all parameters had to be calculated below 50%: interburst interval, burst duration, and number of spike per burst were increased (number of burst per minute decreased) as ρ, and τ_x_ increased. The results of these calculations were shown from 4th row to the last row in Table 3: the values of all calculated percentage errors were between 0 and 47.7%. Here, the results about the number of spike per minute among burst parameters was excluded from Table 3, because all percentage errors in the number of burst per minute were 0.0 and then the values of number of spike during burst would be the same as the values of number of spike per minute.

The results of computer simulation with the values of temperature shown on each panel in Figure 7 are represented as the time series of bursting activity. Although the results of simulation (red solid line) of AP amplitude were not completely optimal, simulation data for number of burst per minute, number of spike during burst, inter-burst interval, burst duration, and interspike interval during burst were well reflected by modified Plant model when these simulation results were compared to the experimental results (blue dotted line).

Therefore, analysis and simulation of the experimental data by our equation model might be helpful for understanding the mechanisms underlying the change of temperature-dependent bursting activity in neurons*.*

## Discussion

In the previous study we analyzed the temperature-dependent change of 14 AP parameters (such as the AP amplitude, membrane potential at the positive peak, ISI, first half and last half of the temperature rising phase, first half and last half of the temperature falling phase, absolute value of the membrane potential at negative peak, absolute value of the maximum slope of the AP during the temperature rising and falling phases, and spiking frequency) in *Aplysia* neurons [[1]]. With these findings, in this study we tried to examine the temperature-dependent change of bursting patterns in pacemaker neuron at abdominal ganglion of *Aplysia juliana.* Furthermore, we attempted to identify the mechanism underlying the temperature dependence of busting by developing an equation model predicting neuronal electrophysiological activity with a function of temperature.

### Temperature-dependent change of bursting patterns

Firstly we conducted real experiments with bursting pacemaker neurons in order to explore the functional relation between temperature and bursting patterns as well as AP parameters. We examined the temperature-dependent activity of bursting pacemaker neurons during several consecutive heating − cooling cycles. We tested the temperature dependencies of AP parameters in bursting pacemaker neurons in the temperature range of 16–30°C. The temperature-dependent properties of AP parameters were analyzed with the selected data shown in Table 1 by using techniques similar to those described in Hyun et al. [[1]].

The values of inter-burst interval, burst duration, and number of spike during burst decreased as the temperature increased, and it was possible to confirm functional properties of temperature dependencies in six burst parameters. When compared to data reported by other research group, Fletcher and Ram analyzed changes of AP parameters of burst-firing neurons during heating by investigating *Aplysia* R15 pacemaker neuron activity in the temperature range of 23 – 37°C; they found that interburst interval, burst duration, and number of spike per burst all decreased within the temperature range of 23 to 32°C [[21]]. We found that our data are consistent with results reported by Fletcher and Ram [[21]] within the temperature range of 23 to 30°C. Furthermore, the present study revealed that the values of ISI and Δt_r1_ decreased and then increased, frequency increased and then decreased, and the parameters Δt_f2_, Δt_1/2_, Δt_r2_, Δtf_1_, and V_np_ all decreased as the temperature was raised. These results were similar to those shown in previously published paper [[1]]. Fletcher and Ram also showed that intra-burst spike broadening and spike height decreased as temperature increased [[21]]. These results were also in good accordance with our experimental results within the temperature range of 23 to 30°C. Because Δt _1/2_ and A_AP,ave_ decreased as temperature increased in the temperature range of 23 to 30°C, as shown in Figure 4D and H: two parameters, spike-broadening and spike height, could be compared with two AP parameters, Δt_AP,1/2_ and A_AP,ave_, respectively. Moreover the present study generated quantitative data regarding the temperature-dependent properties of the other three AP parameters of burst-firing neurons (ISI during burst, number of burst per minute, and number of spike per minute) and nine other AP parameters (ISI, Δt_r1_, Δt_r2_, Δt_f1_, Δt_f2_, θ_1_, θ2, V_np_, and A_AP, max_).

### Mechanism underlying the temperature dependence of busting: choice of the model

Numerous revised model of bursting in R15 [[17]-[19],[28]] have been developed since the mid-1980s. Canavier et al. [[17]] developed the model of bursting in R15 with 10 currents and 11 static variables; 10 currents (the fast Na^+^-current I_Na_, the fast Ca^2+^-current I_Ca,_ the delayed rectifier K^+^-current I_K_, the slow inward Ca^2+^-current I_SI_, the nonspecific cation current I_NS_, the anomalous rectifier current I_R_, the leak current I_L_, K^+^-current I_K_, the Na^+^-Ca^2+^ exchanger current I_NaCa_, the Na^+^-K^+^ pump current I_NaK_, and the Ca^2+^ pump current I_CaP_), and 11 static variables (membrane potential V, intracellular concentration of Ca^2+^ [Ca]_i_, the occupancy of the intracellular Ca^2+^-buffer O_c_, and eight voltage-dependent state-variables (m, h, d, f, n, l, s, b)). The parabolic bursting pattern results from two competing processes, the slow voltage-dependent activation of the slow inward Ca^2+^-current (I_SI_), and the still more slower calcium-dependent inactivation of this same current. Butera et al. [[19]] also developed the models of bursting in R15 with 10 currents and 12 static variables; 10 currents (the fast Na^+^-current I_Na_, the fast Ca^2+^-current I_Ca,_ the delayed rectifier K^+^-current I_K_, the slow inward C^2+^-current I_SI_, the nonspecific cation current I_NS_, the anomalous rectifier current I_R_, the leak current I_L_, K^+^-current I_K_, the Na^+^-Ca^2+^ exchanger current I_NaCa_, the Na^+^-K^+^ pump current I_NaK_, and the Ca^2+^ pump current I_CaP_), and 12 static variables (membrane potential V, intracellular concentration of Ca^2+^ [Ca^2+^]_i_, intracellular concentration of cAMP [cAMP], the occupancy of the intracellular Ca^2+^-buffer O_c_, and eight voltage-dependent state-variables (m, h, d, f, n, l, s, b)). The slow inward Ca^2+^ current (I_SI_) in this model is the key current responsible for leading to bursting phenomena.

In addition to these models, Bertram [[18],[29]] developed a mathematical model of bursting neuron R15 including 8 currents and 11 static variables; 8 currents (excitatory sodium current I_Na_, excitatory calcium current I_Ca_, inhibitory potassium current I_K1_ and I_K2_ , small leakage current I_L_, calcium current which initiate the burst I_NSR_, and cation-nonspecific current I_D_, and a potassium current which is activated only near the potassium equilibrium potential I_R'_) and 11 static variables (membrane potential V, intracellular concentration of Ca^2+^ c, and nine voltage-dependent state-variables (m, h, n, j, q, y, μ, r, x)). Two subthreshold burst currents, the calcium current which initiate the burst I_NSR_ and the cation-nonspecific current I_D_, induce the busting oscillation.

To mathematically clarify the temperature-dependent changes of bursting patterns in burst-firing neurons, it was suggested to perform computational simulations of these phenomena by using a proper model composed of equations with temperature-dependent scaling factors.

**Figure 8:**
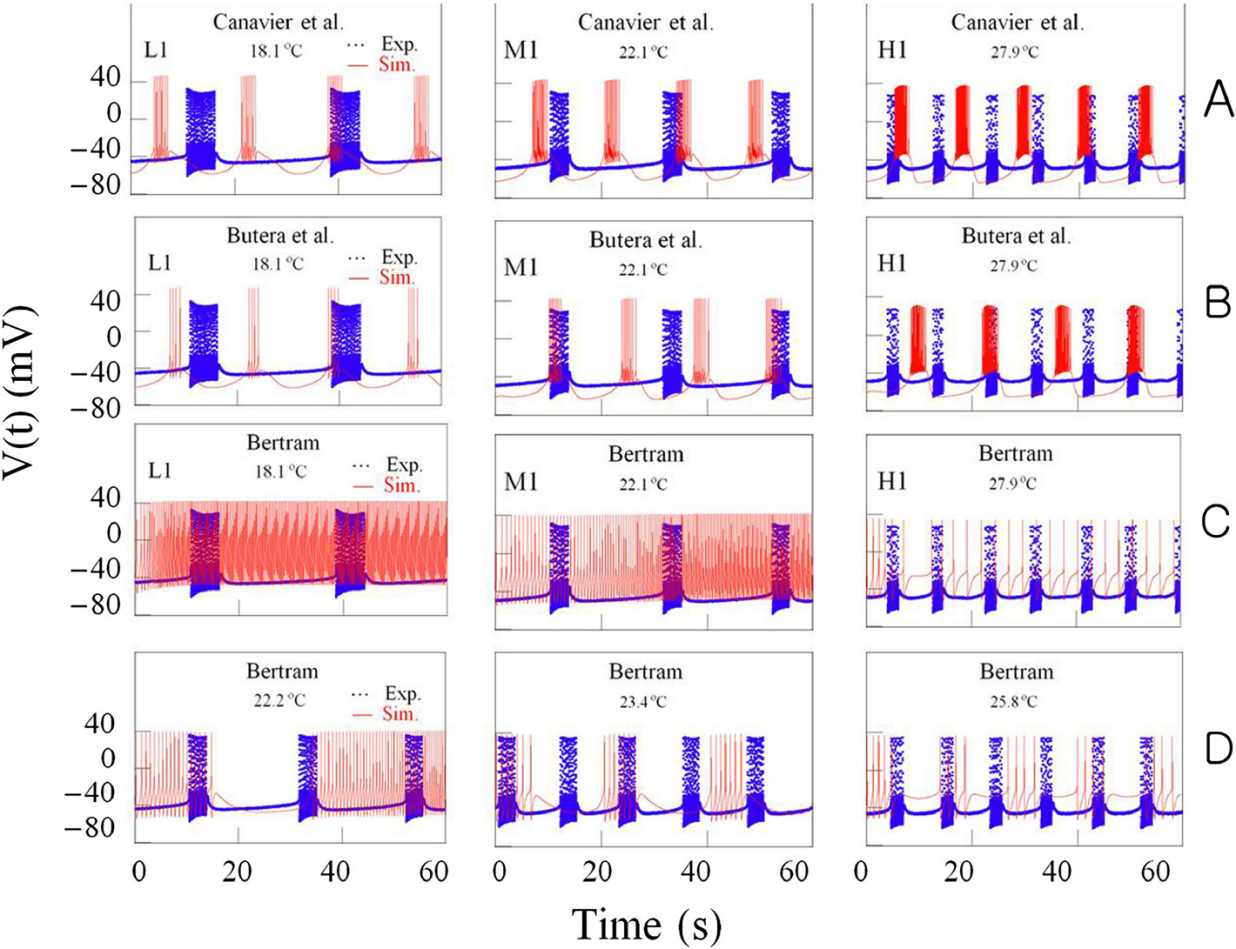
**Time series of bursting activity drawn by using the data for the experiments A and computer simulations at different temperatures.** Experimentally recorded spike trains (blue, dotted line) and the result from computer simulations of modeling (red, solid line) are compared. The results from simulation using the equations developed by Canavier et al. [[17]] and modified by us with temperature-dependent scaling factors at the temperatures, 18.1°C, 22.1°C and 27.8 are shown on panels L1, M1, and H1 respectively, in the upper traces **(A)**. Those using the models revised by Butera et al. [[19]] and Bertram [[18]] and modified by us with the same temperature dependent scaling factors are shown in the intermediate traces **(B,C)**. The comparison of those using the equations revised by Bertram and modified by us are shown in the lowest panels **(D)** at the temperatures 22.2°C, 23.4°C and 25.8°C with small range of temperature changes.

We have tried to conduct computational simulations by using modified models: in modifying the models that were developed by Canavier et al. [[17]] and Butera et al. [[19]], the maximal conductances of currents ${\bar{g}}_{i}$ were multiplied by temperature-like scaling factor for currents $\rho \left(T\right)\;({\bar{g}}_{i}\times \rho \left(T\right)={\bar{g}}_{i}\times 1.3^{\frac{{T‐T}_{0}}{10{}^{\circ}C}}$, i = Na, Ca, K, SI, NS), and the relaxation time constant T_j_ were multiplied by temperature-like scaling factor for ionic kinetics $T_{j}\left(\left(1/T_{j}\right)\times \phi \left(T\right)\right)=\left(1/\tau_{j}\right)\times 3^{\frac{{T‐T}_{0}}{10{}^{\circ}C}}$, j = m, h, d, f, n, l, s, b). In order to obtain computer simulation of temperature-dependent bursting patterns, it was necessary to select three good data files obtained from experiment A at different temperatures; 18.1°C, 22.1°C, and 27.9°C. The figures drawn by using these data files were expressed by symbols L1, M1 and H1, respectively, shown on the panels at Figure 8A and B. After temperature values shown on these panels should be substituted for T in the temperature-dependent scaling factors, ρ(T) and ϕ(T) involved in these modified models (T_0_ = 23°C), we could get these figures drawn in red solid lines and these were shown on these panels. In the results of simulations with these modified models, it could be found that burst duration and number of spike during burst increased as temperature increased. But these results were not consistent with our experimental results. So, although the model presented by Canavier et al. [[17]] and Butera et al. [[19]] were based on experimental data to a great extent, it might be thought that these models are not appropriate for investigation about the ionic mechanism underlying the temperature-dependent change in bursting pattern from experiments with bursting pacemaker neurons in the abdominal ganglia of *Aplysia juliana*.

In a similar way, we could get three figures shown on Figure 8C by simulation using the modified models that were developed by Bertram [[18]]. Although the fact that the values of burst duration and number of spike during burst that were obtained from simulation by using this modified model decreased as temperature increased was consistent with our experimental results in a small temperature range, it was very difficult to simulate the temperature-dependent changes of bursting patterns in a large temperature range; from 18.1°C to 27.9°C. So we tried to simulate temperature-dependent bursting patterns in a small temperature range from 22.2°C to 25.8°C and the results were shown on Figure 8D. But these results were also not consistent with our experimental results. Namely, the values of interspike interval during burst obtained from simulations were larger than those got from experiments, and these values could not be reduced: the values of the other bursting parameters might be given rise to inconsistencies with our experimental results. Thus, it might be thought that this model was not also appropriate for investigation about that.

Any way, it was shown that that I_K(Ca)_ is not involved in burst termination in *Aplysia* bursting neurons Kramer and Zucker [[30]]. Even though calcium-activated potassium channels are located in the soma of R15 neuron, it was not necessarily proved that this channel was important for bursting pattern. Next, we have to discuss on the following question: are there any other experimental evidences suggesting that I_K(Ca)_ is involved in bursting pattern of *Aplysia* neurons, particularly R15 neuron? Although we didn’t yet find any one of research papers that were published after year 1985, it might be necessary to discuss whether the figures that we have suggested in this paper could be given us any chance to run back over the experimental evidence on it.

**Figure 9:**
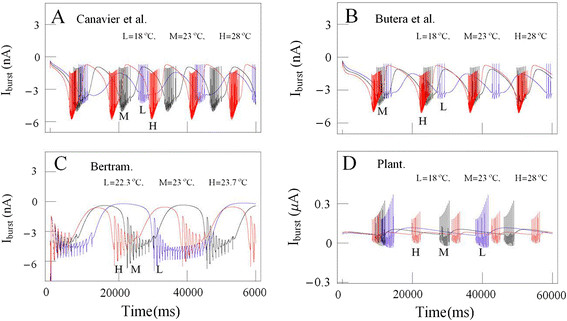
**Comparison of temperature-dependent subthreshold current activities. (A)** Time series of temperature-dependent subthreshold current activities (ρ(T)∙I_SI_) simulated by using the results based on the model developed by Canavier et al. [[17]] at temperature values 18°C (L, blue lines)), 23°C (M, black lines), and 28°C (H, red lines) are shown together. **(B)** Those (ρ(T)∙I_SI_) obtained from simulation data at the same temperature values using the model made by Butera et al. [[19]] are shown together. **(C)** Those (I_D_ + ρ(T)∙I_NSR_) taken from simulation data at temperature values 22.3°C, 23°C, and 23.7°C using the model made by Bertram et al. [[18]] are shown together. **(D)** Those (ρ(T)∙(I_Ca_ + I_K(Ca)_)) obtained from simulation data at temperature values 18°C, 23°C and 28°C using the model made by Plant [[15]] are shown together.

Now, it might be necessary to compare the temperature-dependent changes in burst firing patterns of subthreshold currents (or burst currents) involved in Plant model with those included in the other models. Time series of temperature-dependent subthreshold current activities (ρ(T)∙I_SI_) simulated by using the equation of slow inward calcium current based on the model developed by Canavier et al. [[17]] at temperature values 18°C, 23°C, and 28°C are shown together in blue lines (L), black lines (M), and red lines (H), respectively on panel A in Figure 9. Those (ρ(T)∙I_SI_) and (ρ(T)∙(I_Ca_ + I_K(Ca)_)) obtained from simulation data by using the model made by Butera et al. [[19]] and Plant [[15]] at the same temperature values are shown together on panel B and D in Figure 9, respectively. Those (I_D_ + ρ(T)∙I_NSR_) taken from simulation data calculated by using the model made by Bertram [[18]] at temperature values 22.3°C, 23°C, and 23.7°C are shown together on panel C in Figure 9. Next, it might be wanted to calculate the maximum values of temperature-dependent burst currents underlying the hyperpolarization of the inter-burst intervals, and then compare the values that were calculated by using Plant developed model with those by using the other models. The maximum values of these calculated by using Plant model at temperature values 18°C, 23°C, and 28°C were 0.28 μA, 0.12 μA, and 0.081 μA, respectively. The maximum values calculated by using Canavier et al. (Butera et al.) developed model at the same temperature values were −1.62 nA, −0.9 nA, and −0.75 nA (−1.5 nA, −1.1 nA, and −0.77 nA), respectively. The maximum values calculated by using the model that Bertram developed at the temperature values 22.3°C, 23°C, and 23.7°C were −0.18 nA, −0.38 nA, and −0.58 nA, respectively. These absolute maximum values calculated by using the models that Plant, Canavier, and Butera developed decreased as temperature increased, but those calculated by Bertram developed model increased as temperature increased. But more detailed analysis of these data remained as a challenge for future study.

Anyway, these facts might show that it was hard to clarify mathematically the temperature-dependent changes of bursting patterns at burst-firing neurons of *Aplysia juliana* with these three models involving many currents and a large number of static variables. So, it was necessary to investigate much simpler model with small number of currents and static variables such as Plant model.

At this stage, it might be useful to remind that temperature-dependent impulse patterns of mammalian cold receptors could be well simulated by using nonlinear differential equations involving I_K(Ca)_. Different types of impulse patterns of mammalian cold receptors can be observed as a function of skin temperature: irregular and less frequent burst discharges, regular and frequent bursting patterns, and irregular single spike patterns are observed from low to high temperatures. These patterns could be simulated by Braun et al., and the Huber-Braun cold receptor model has been described in detail with 5 currents and 5 static variables (membrane potential and four voltage-dependent state-variables) [[26],[27],[31]-[36]]. This model consisted of two minimal sets of ionic conductances operating at different voltage levels with different delays and the leakage current. The first two voltage-dependent currents that generate the action potentials mean depolarizing current I_Na_ and repolarizing current I_K_. The next two voltage-dependent slow currents that generate subthreshold potential oscillations were slow depolarizing noninactivating Na^+^-current I_Nap_ and slow repolarizing Ca^2+^-dependent K^+^-current I_K(Ca)_ with voltage dependent activation of Ca^2+^-current. The temperature dependences were given by temperature-like scaling factors for the maximum conductances and the time constants, with reference temperature T_0_ = 25°C. Q_10_ of 3.0 for the activation variable and Q_10_ of 1.3 for temperature dependences of maximum conductances were chosen.

Now, it would be necessary to look into another simple model to investigate the mechanisms of temperature-dependent changes of bursting patterns which share a few similarities with mammalian cold receptors. Plant model consisted of 5 currents and 6 static variables: 5 currents (fast sodium current I_Na_, fast potassium current I_K_, inward calcium current I_Ca_, calcium-activated potassium current I_K(Ca)_, and leakage current, I_L_) and 6 static variables (membrane potential, intracellular concentration of Ca^2+^, and four voltage-dependent state-variables). There are two fast and slow processes in this model. The fast process had three components: the activation and inactivation variables for Na^+^ channels “m” and “h”, respectively, and the activation of K^+^ channels n. The slow process had two components: a slow conductance for Ca^2+^-current “X”, and intracellular free calcium concentration “Ca”. Not only Plant model had a simple structure, but also theoretical analyses of Plant’s model were performed already [[16],[37],[38]].

However, it is not easy to justify the reason of our choice of Plant model, because some papers excluded I_K(Ca)_ as a key bursting current in R15 and focused on the inward current with slow activating and inactivating components. Plant model was based on the works of Gorman and Thomas [[39]], who demonstrated that I_K(Ca)_ was linearly dependent on increasing concentration of intracellular calcium ions (Ca^2+^) injected into the cytoplasm and expected to be activated during a burst. Chay [[40]] constructed mathematical model applicable to the *Aplysia* bursting neurons with involving the properties of Ca^2+^-activated K^+^ channel: intracellular Ca^2+^ concentration increases gradually during activity to levels where it activates the I_K(Ca)_ [[10]]. Although Kramer and Zucker [[30]] concluded that I_K(Ca)_ did not play a role in burst termination in bursting neurons of *Aplysia*, Adams and Levitan [[41]] did not exclude a role for I_K(Ca)_ in the repolarization of action potential. But they asked the question: why a long-lasting I_K(Ca)_ did not be activated by action potential? The plausible answer was the amount of calcium ions that entered into the cell during the activity of action potential was insufficient to generate it. Even if Canavier et al. [[17]] developed the model without I_K(Ca),_ it was suggested as a limitations of this model that more experimental data of I_K(Ca)_ were required for further investigation. Bertram [[18]] revised the model with I_K(Ca)_ conductance depended only on the constant calcium concentration of the soma. Besides this, it has been known that the calcium activated potassium channels are located in R_15_ soma [[42]] and electrode was inserted into the soma of the cell in the abdominal ganglion of *A. juliana* to measure the membrane potentials in our experiments. Thus, we cannot underestimate the impact of I_K(Ca),_ but we did not here want to claim the quantitative application of Plant model, rather, we used it fr understanding the mechanism underlying the temperature dependence of busting patterns by computer simulations: it might be challenging to work later with a Rinzel and Lee’s model based on the hypothesis that it is necessary for burst generation to consider an inward current which slowly inactivates as Ca^2+^ accumulates during the burst to the exclusion of I_K(Ca)_ [[16]]. By making a comparison between experimental data and simulation results calculated with modified Plant equations, it was suggested that the mechanism underlying temperature-dependent bursting patterns of bursting pacemaker neurons at abdominal ganglion of *Aplysia juliana* might be derived from temperature-like scaling factors ρ(T) and ϕ(T) for the maximum conductances and the time constants, respectively; together with reference temperature T_0_ = 23°C, Q_10_ of 3.0 for the activation variable, and Q_10_ of 1.3 for temperature dependences of maximum conductances.

Taken together, it was suggested that a modified Plant model could be used to simulate the temperature-dependent bursting activity of bursting pacemaker neurons in the abdominal ganglia of *Aplysia juliana* and to unravel the mechanism of temperature dependence in bursting patterns.

### Nomenclature

A_AP_, action potential amplitude

A_AP, ave_, averaged action potential amplitude

A_AP, max_, maximum action potential amplitude

Ca, slow change in intracellular free calcium concentration

Frequency, spontaneous firing frequency (=1/ISI)

ISI, interspike interval (=Δt_r1_ + Δt_AP, 1/2_ + Δt_f2_)

I_Ca_, slow inward calcium current

I_K_, fast potassium current

I_K(Ca)_, calcium-activated potassium current

I_L_, leak current

I_Na_, fast sodium current

T_0_, reference temperature (=23°C)

V, membrane potential

V_Ca_, reversal potential for calcium current

V_K_, reversal potential for potassium current

V_L_, reversal potential for leak current

V_Na_, reversal potential for sodium current

V_np_, membrane potential at the negative peak

V_pp_, membrane potential at the positive peak

Δt_r1_, first half of the rising phase of AP

Δt_r2_, last half of the rising phase of AP

Δt_f1_, first half of the falling phase of AP

Δt_f2_, last half of the falling phase of AP

Δt_AP, 1/2_, AP half-width duration (=Δt_r2_ + Δt_f1_)

θ_1,_ inverse tangent of the ratio of half A_AP_ to Δt_r2_

θ_2_, inverse tangent of the ratio of half A_AP_ to Δt_f1_

${\bar{g}}_{\mathrm{Ca}}$, maximal conductance for calcium ion current

${\bar{g}}_{K}$, maximal conductance for potassium ion current

${\bar{g}}_{L}$, maximal conductance for leak current

${\bar{g}}_{\mathrm{Na}}$, maximal conductance for sodium ion current

*h,* voltage-dependent inactivation variable for sodium channel; h_∞_ = steady state value of inactivation h

*m,* voltage-dependent activation variable for sodium channel; m_∞_ = steady state value of activation m

*n,* activation variable for potassium channel; n_∞_ = steady state value of activation n

μ_m_, constant value involved in the defining equation for m_∞_

μ_h_, constant value involved in the defining equation for h_∞_

μ_n_, *v*_n_, constant values involved in the defining equation for n_∞_

α, β, γ, δ, constant values involved in the defining equation for steady state values of activation (inactivation) variables and relaxation constants

1/λ, maximal relaxation time constant of h and n

${\bar{\tau }}_{n}$, maximal relaxation time constant of n

τ_h,_ relaxation constant of h

τ_χ,_ relaxation constant of χ

τ_n,_ relaxation constant of n

χ, slowly activating conductance for calcium current; χ_∞_ = steady state value of activation χ

ρ^−1^, estimation for the time-constant of the Ca-equation

ρ(T), temperature-dependent scaling factor; $\rho \left(T\right)\equiv 1.3^{\frac{{T‐T}_{0}}{10^{{}^{\circ}C}}}$

ϕ(T), temperature-dependent scaling factor; $\phi \left(T\right)\equiv 3^{\frac{{T‐T}_{0}}{10^{{}^{\circ}C}}}$

I_SI,_ slow inward Ca^2+^-current

I_NS,_ nonspecific cation current

I_R,_ anomalous rectifier current

I_NaCa,_ Na^+^-Ca^2+^ exchanger current

I_NaK,_ Na^+^-K^+^ pump current

I_CaP,_ Ca^2+^ pump current

I_K1,_ inhibitory potassium current

I_NSR,_ calcium current which initiate the burst

I_D,_ cation-nonspecific current

I_R,_ potassium current which is activated only near the potassium equilibrium potential

I_Nap,_ slow depolarizing noninactivating Na^+^-current

## Competing interests

The authors declare that they have no competing interests.

## Authors’ contributions

N.G.H. carried out electrophysiological experiments, performed computer simulation of experimental data, and drafted the manuscript. K.H.H. and K.B.H. participated in computer programming for simulation of experimental data. K.L. drafted and revised the manuscript. All authors read and approved the final manuscript.
